# Children—Their Diseases and Treatment

**Published:** 1872-11

**Authors:** 


### Miscellaneous.

### Children—Their Diseases and Treatment.

The following is from an address by Dr. MATTOCKS, in the *Transactions of the Minnesota State Medical Society*:—

A very common error we often fall into is the generalization of the diseases of children; for instance, the treatment of teething, and its many complications, as a special disease.

This brings us to a brief consideration of the treatment of children.

Advanced medical science advises but little medicine in the treatment of children. Advanced ignorance admits no medicine at all. As a general rule the acute endemic diseases of infancy are traceable to some known cause; such as scanty or improper food—else exposure to the changes and vicissitudes of the weather; or, to put our proposition differently, adult diseases occur, children's sicknesses are caused. Taking this view of the case, the indication would be to protect children from the ignorance of their nurses, to demonstrate to them, if possible, the cause of the present attack, that they may in the future be on their guard against similar exposures.

In our *practice* with adults, we often neglect to trace out the cause of their disease. In many instances it is unnecessary. With children, however, the case is very different. Here we must find, oftentimes, our indication for treatment from the cause of the disease. For instance, in diarrhœa. With the adult, the simple fact of diarrhœa may be sufficient. Our duty is to check it. With the infant, on the contrary, we must know what caused the diarrhœa, whether it be irritating food, an insufficiency of food, or whether it be simply the result of reflex irritation.

The diarrhœas of childhood, as a general thing, are the result of improper food. This leads to the inquiry, what is the proper food for an infant? It is certainly strange that we should ever find it necessary to ask this question, what food shall we give a baby? Give it milk, of course—breast milk, if possible, if not, good cow's milk. But are you sure about this, you ask—better men than you have advised differently. Another says, I have fed my children with an artificial preparation, and they thrive on it. Yes, says another, and even better than upon breast milk. To this we answer, we have seen children thrive in squalor, filth, and exposure, but where one has lived through this ordeal of uncleanness, a dozen have died by reason of it.

Most of the writers upon the diseases of children, and all the discoverers of artificial food for infants, dwell in large cities where it is impossible to obtain good cow's milk, and rather than use an impure article, they are forced to resort to other means.

It does not necessarily follow, however, that because Prof. Meigs has found it necessary in Philadelphia to modify the diet of his little patients—when good food cannot be obtained—that you and I must do the same, living in a land where good milk is abundant, any more than we should send our sick babies to Fairmount Park, in Philadelphia, because the physicians of Philadelphia do it. Because Baron Leibig found it necessary to compound a food for his little grandson, must we use the same for everybody else's little grandsons? If good breast-milk cannot be obtained, I think we should establish a rule that good cow's milk, properly diluted, should be its substitute—that is, if good cow's milk can be obtained, of course insisting that milk from one cow should be used. In some instances, few we think, this food does not seem to agree with our patients; perhaps we do not sufficiently dilute it, or may be the milk is not fresh (we should bear in mind that nature furnishes the infant milk fresh from the breast), or that the utensils are not clean, or some other reason may exist, which patient and judicious investigation may remove.

A few pages might be spent with fitness in discussing the clothing of children, or rather the want of it. The physician is culpable who does not attend to the clothing of his little patients, or rather call the mother's attention to the matter, from time to time.

We remarked that the diseases of children were not necessarily peculiar. This is true of the treatment of children. They must be treated symptomatically. It seems to us that children suffer more than adults when sick, just as nervous people suffer more when sick than the phlegmatic. Besides, the alimentary canal and the brain seem to be the seat of diseases more often with the little ones, and our personal experience teaches us that where either the one or the other is involved, much suffering ensues.—*Medical and Surgical Reporter.*

